# Bisphenols and Oxidative Stress Biomarkers—Associations Found in Human Studies, Evaluation of Methods Used, and Strengths and Weaknesses of the Biomarkers

**DOI:** 10.3390/ijerph17103609

**Published:** 2020-05-21

**Authors:** Inger-Lise Steffensen, Hubert Dirven, Stephan Couderq, Arthur David, Shereen Cynthia D’Cruz, Mariana F Fernández, Vicente Mustieles, Andrea Rodríguez-Carillo, Tim Hofer

**Affiliations:** 1 Department of Environmental Health, Section of Toxicology and Risk Assessment, Norwegian Institute of Public Health, PO Box 222 Skøyen, N-0213 Oslo, Norway; inger-lise.steffensen@fhi.no (I.-L.S.); hubert.dirven@fhi.no (H.D.); 2 Départment “Adaption du Vivant“, Physiologie Moléculaire et Adaptation, Muséum National d’Histoire Naturelle, UMR 7221 MNHN/CNRS, 7 rue Cuvier, 75005 Paris, France; stephan.couderq@mnhn.fr or; 3 Univ Rennes, Inserm, EHESP, Irset (Institut de Recherche en Santé, Environnement et Travail)-UMR_S 1085, F-35000 Rennes, France; arthur.david@ehesp.fr (A.D.); or shereencynthia@gmail.com (S.C.D.); 4 Department of Radiology and Physical Medicine, and Center for Biomedical Research (CIBM), University of Granada, 18016 Granada, Spain; marieta@ugr.es (M.F.F.); vmustieles@ugr.es (V.M.); andrearc@ugr.es (A.R.-C.); 5 Consortium for Biomedical Research in Epidemiology & Public Health (CIBERESP), 28029 Madrid, Spain

**Keywords:** adverse outcome pathway (AOP), analytical methods, antioxidant, bisphenol F (BPF), bisphenol S (BPS), effect biomarker, HBM4EU, 4-hydroxy-2-nonenal-mercapturic acid (HNE-MA), reactive nitrogen species (RNS), reactive oxygen species (ROS)

## Abstract

Bisphenols, particularly bisphenol A (4,4′-(hexafluoroisopropylidene)-diphenol) (BPA), are suspected of inducing oxidative stress in humans, which may be associated with adverse health outcomes. We investigated the associations between exposure to bisphenols and biomarkers of oxidative stress in human studies over the last 12 years (2008‒2019) related to six health endpoints and evaluated their suitability as effect biomarkers. *PubMed* database searches identified 27 relevant articles that were used for data extraction. In all studies, BPA exposure was reported, whereas some studies also reported other bisphenols. More than a dozen different biomarkers were measured. The most frequently measured biomarkers were 8-oxo-7,8-dihydro-2′-deoxyguanosine (8-OHdG), 8-iso-prostaglandin F2α (8-isoprostane) and malondialdehyde (MDA), which almost always were positively associated with BPA. Methodological issues were reported for MDA, mainly the need to handle samples with caution to avoid artefact formation and its measurements using a chromatographic step to distinguish it from similar aldehydes, making some of the MDA results less reliable. Urinary 8-OHdG and 8-isoprostane can be considered the most reliable biomarkers of oxidative stress associated with BPA exposure. Although none of the biomarkers are considered BPA- or organ-specific, the biomarkers can be assessed repeatedly and non-invasively in urine and could help to understand causal relationships.

## 1. Introduction

**Figure 1:**
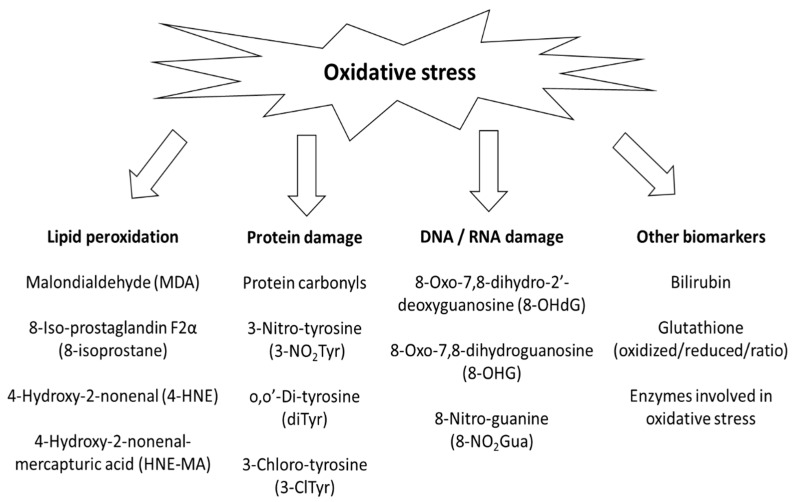
Commonly measured biomarkers of oxidative stress after BPA exposure in humans.

Bisphenol A (4,4′-(hexafluoroisopropylidene)-diphenol) (BPA) is an important monomer in the production of polycarbonate and epoxy plastics, but it is also used in various other applications. In polyvinylchloride (PVC) plastic, BPA acts as an antioxidant [1]. Due to its high production volume and migration from plastics, human exposure to BPA is widespread. BPA is suspected for endocrine (xenoestrogen) disruptive effects in humans and in wild-life (see European Chemical Agency, link: (https://echa.europa.eu/-/seven-new-substances-added-to-the-candidate-list-entry-for-bisphenol-a-updated-to-reflect-its-endocrine-disrupting-properties-for-the-environment).The European Food Safety Authority (EFSA) has established an acceptable daily intake (ADI) of 4 µg/kg body weight per day [2]. However, studies in animals and cell cultures suggest that BPA can induce oxidative stress [3] by generating reactive oxygen species (ROS), such as phenoxyl radicals (RO^•^) [4], the superoxide anion (O_2_^•−^), and hydrogen peroxide (H_2_O_2_), presumably through the formation of a reactive *o*-quinone radical [5,6] during its metabolism. Endogenously produced ROS can inflict damage to nucleic acids, proteins and initiate lipid peroxidation in cellular membranes [7], leading to the formation of oxidized products that, after export into the circulation and excretion into urine, can be used as biomarkers of oxidative stress, see Figure 1.

Of the major bases in nucleic acids, guanine is the easiest oxidized base [8]. As an oxidized nucleoside in the cytosol, inside nuclear and mitochondrial DNA, 8-oxo-7,8-dihydro-2′-deoxyguanosine (8-oxodGuo or 8-OHdG) is one of the predominant forms of ROS- and free radical-induced lesions in DNA and has been widely recognized as a biomarker of oxidative stress [7,8]. Various studies show that urinary 8-OHdG is increased during certain exposures, e.g., smoking and irradiation, and conditions/diseases such as inflammation. Traditionally, urinary 8-OHdG has been used to estimate DNA damage in humans after exposure to cancer-causing agents, such as tobacco smoke, asbestos fibers, heavy metals, and polycyclic aromatic hydrocarbons [9]. Similarly, oxidation of RNA, which is generally more abundant and less protected than DNA, leads to the formation of 8-oxo-7,8-dihydroguanosine (8-oxoGuo or 8-OHG) [10].

Oxidants can also initiate lipid peroxidation, an oxygen (O_2_)-driven process generating oxidized lipids and various reactive aldehydes, such as malondialdehyde (MDA) and 4-hydroxy-2-nonenal (4-HNE) that can react with cellular macromolecules, including proteins (can result in protein carbonyls) and DNA, leading to the formation of adducts [7]. As urinary biomarkers of lipid peroxidation, 8-iso-prostaglandin F_2α_ (8-isoPF_2α_, 15F_2t_-isoprostane or 8-isoprostane) derived from arachidonic acid (Ω-6) and MDA, are commonly measured. The reactive lipid peroxidation product 4-HNE, which stems from Ω-6 fatty acids, can react with glutathione (GSH) to form 4-hydroxy-2-nonenal-glutathione (HNE-GSH), which is metabolized into 4-hydroxy-2-nonenal-mercapturic acid (HNE-MA) and excreted into urine [11].

Plasma sulfhydryl (SH) groups are also susceptible to oxidative damage, which can lower the available SH groups. Essentially all blood plasma SH groups are protein-associated, although plasma also contains low amounts of the reduced GSH form [12]. Total thiols represent all available SH groups in a sample. GSH participates in enzymatic removal of peroxides, forming oxidized glutathione (GSSG), and GSSG or GSSG/GSH in serum are sometimes used as biomarkers for oxidative stress. During the continuous turnover of red blood cells, catabolism of hemoglobin results in a steady supply of bilirubin, which is thought to be a major antioxidant in blood [7].

Reactive nitrogen species (RNS), e.g., peroxynitrite anion (ONOO^−^) and nitrogen dioxide (NO_2_), can react with biomolecules to form adducts, e.g., 8-nitro-guanine (8-NO_2_Gua) in nucleosides. ONOO^−^ is formed through the reaction of nitric oxide (NO^•^) with O_2_^•−^. Similarly, RNS can react through nitration with the phenolic amino acid tyrosine, forming 3-nitro-tyrosine (3-NO_2_Tyr). Neutrophils can through myeloperoxidase release the oxidant hypochlorous acid (HOCl), which can react with tyrosine, forming 3-chloro-tyrosine (3-ClTyr). Oxidation of two nearby tyrosines within proteins can lead to the formation of *o*,*o*’-di-tyrosine (diTyr). Subsequent protein proteolysis releases these modified amino acids, which are excreted into the urine.

Protein carbonyls, e.g., oxidized amino acids within proteins and/or aldehydes having reacted with proteins, gene/protein expression profiles (omics-related), and genetic polymorphism, e.g., variants in antioxidant enzyme coding genes, are also used as biomarkers of oxidative stress [7]. As a consequence of increased oxidative stress, oxidized products (e.g., 8-OHdG) and expression of protective antioxidant enzymes are expected to increase, whereas antioxidants will be consumed and are expected to decrease. Some oxidative stress effect biomarkers, e.g., 8-OHdG, MDA, and 8-isoprostane, have been studied for decades and are mentioned in several adverse outcome pathways (AOPs) (https://www.aopwiki.org). However, their use as biomarkers of effect in environmental epidemiological studies investigating the effects of non-persistent chemicals is more recent.

In a comprehensive review of the BPA literature, we identified various types of effect biomarkers associated with bisphenol exposure in human studies for the years 2008‒2017 [13] as part of the European Union-funded Horizon 2020 HBM4EU project (website https://www.hbm4eu.eu/). Given the wide scope of the previous work, it was not possible to analyze in depth all the oxidative stress biomarkers identified, perform comparisons of analytical methods or discuss potential mechanisms. Therefore, the present paper has been extended to years 2018‒2019 to investigate in more detail the use of oxidative stress biomarkers in human studies with the aim of also investigate the strengths and weaknesses of these oxidative stress biomarkers as effect biomarkers of bisphenol exposure. In addition, the various methods used for measurements of these biomarkers are discussed.

## 2. Materials and Methods

Terms used in *PubMed* database searches for bisphenols (BPs) and the six health endpoints (‘behavior, neurodevelopment, and neurodegeneration’, ‘cancer’, ‘endocrine diseases’, ‘immune system and allergy’, ‘obesity, metabolic disorders, and cardiovascular diseases’ and ‘reproductive diseases’) are listed as Table A1 in Appendix A and were selected by a teamwork HBM4EU effort. Searches were limited to human studies and full-text papers using available filters. Searches took place at the turn of the year 2017‒2018 (limited to years 2008‒2017) [13]. On 3 January, 2020, the previous search was specifically updated for oxidative stress biomarkers until the years 2018‒2019. Thus, the current study covers human references focused on bisphenol-related oxidative stress biomarkers published between 2008 and 2019. Oxidative stress terms were not specifically searched for, but papers mentioning oxidative stress-related measurements in their titles and abstracts were read in detail to identify oxidative stress effect biomarkers. Only papers that included results on biomarkers of effects of bisphenols A, AF, F, and S were included, but also other bisphenols are mentioned when measured in such papers.

Review papers, ex vivo–in vitro and in vitro studies were excluded. Additionally, since some apparently relevant articles were detected that had not been identified using the search terms for the six health endpoints, a complementary *PubMed* search using the broad search terms “bisphenol AND oxidative stress AND biomarker”, limited to ‘humans’, was performed on 27 January, 2020, without a time limit.

## 3. Results

### 3.1. Literature Search Results

In the first PubMed search for the years 2008‒2017, 14 human articles related to bisphenols and biomarkers of oxidative stress were found [13]. In the second search on 3 January, 2020, using the same search terms, 10 additional relevant articles for the years 2018‒2019 were found, thus, there seems to be an increasing trend in the number of papers on this topic. The complementary broad search performed at the end of January 2020 resulted in three additional relevant articles, therefore, in total, 27 articles on bisphenols and biomarkers of oxidative stress were identified.

### 3.2. Associations between Bisphenol Exposure and Oxidative Stress Biomarkers in Human Studies

The 27 articles identified on bisphenols and oxidative stress biomarkers were heterogeneous, regarding study design, the number of participants, healthy vs. patients, oxidative stress biomarker studied, analytical methods, and results. Therefore, it was not considered appropriate to perform a meta-analysis. Only seven studies presented data on adverse health outcomes, including non-alcoholic fatty liver disease (NAFLD), attention deficit hyperactivity disorder (ADHD), autism (two papers), chronic obstructive pulmonary disease (COPD), infertility, and gynecological complaints (Table 1). The other papers were grouped into the following categories: pregnant women and their fetuses/newborns (six papers), children and adolescents (four), adults (seven), elderly (two), and based on residence area (one). Twenty papers were focused on BPA only, whereas seven papers also included from 1 to 8 additional bisphenols. For each article, the results related to biomarkers of oxidative stress were described in the following text and summarized in Table 1.

#### 3.2.1. Diseases and Adverse Health Conditions

In an Italian prospective study by Dallio et al., 2018 [14], non-alcoholic fatty liver disease (NAFLD) patients (*n* = 60) were enrolled and further divided into two groups based on absence or presence of necroinflammation and fibrosis at histology: non-alcoholic fatty liver (NAFL) patients (*n* = 30; 54 ± 13 years) with simple lipid accumulation in the hepatocytes, and non-alcoholic steatohepatitis (NASH) patients (*n* = 30; 57 ± 11 years), as well as healthy sex-matched controls of both genders (*n* = 60; 56 ± 8 years). Among the patients, some also had type 2 diabetes mellitus (T2DM); the numbers seem to be 11 NAFL and 16 NASH patients (numbers in the article’s text and table do not match). The levels of BPA were significantly higher in the NAFLD patients compared to controls in both 24 h urine (*p* < 0.0001; creatinine-adjusted) and plasma (*p* < 0.0001). The plasma BPA levels were significantly higher in NASH patients than in NAFL patients (*p* = 0.041), independent of the presence of T2DM. There was also a significant association between BPA plasma levels and the degree of inflammation evaluated according to Kleiner et al., 2005 [15] as absent, low, moderate, or severe. On the other hand, urinary BPA levels were lower in NASH versus NAFL patients, although this difference was not statistically significant (*p* = 0.201). The NAFLD patients showed higher values of serum 2-thiobarbituric (TBA) reactive substances (TBARS) (colorimetric analysis, no separation step used) compared to controls (*p* < 0.01). Moreover, NASH patients showed higher TBARS levels in comparison to NAFL patients, but this result was not statistically significant (*p* = 0.104). Serum superoxide dismutase (SOD) and catalase (CAT) activities were greater in the NAFLD group than in controls (*p* < 0.01), but the differences between NAFL and NASH patients were not statistically significant (*p* = 0.081). An eventual association between BPA and the biomarkers of oxidative stress (TBARS, SOD, and CAT activities) was not statistically tested, but higher BPA levels in NAFLD patients were associated with increased TBARS levels as well as higher SOD and CAT activities (all in serum). After a BPA-free diet for 1 month, NAFLD patients showed a significant reduction in BPA plasma levels (*p* = 0.016), without a significant reduction in urine levels (*p* = 0.221). This resulted in lower TBARS levels, and lower SOD and CAT activities, in comparison to before the intervention, but not in a statistically significant manner.

In a Chinese case-control study, Li et al., 2018 [16] measured urinary (morning spot sample) BPA and 8-OHdG levels in 6‒12-year-old children diagnosed with attention deficit hyperactivity disorder (ADHD) (*n* = 215) and healthy children (*n* = 253) living in Guangzhou. Levels were creatinine-adjusted. The ADHD group had significantly higher urinary BPA (*p* < 0.001) and 8-OHdG (*p* < 0.001) levels vs. controls, and 8-OHdG correlated positively with BPA (r = 0.257, *p* < 0.001). Younger (6‒9 years) children had higher urinary BPA levels than older (10‒12 years) children.

An Egyptian case-control study by Metwally et al., 2018 [17] investigated the role of BPA in children with autism spectrum disorders (ASD) (*n* = 49; mean age 5.95 ± 1.91 years) and in age- and sex-matched controls (*n* = 40; mean age 5.33 ± 2.28 years). BPA and oxidative stress biomarker analyses were performed in serum. The results showed that both BPA (*p* = 0.025) and 8-OHdG (*p* = 0.0001) were significantly higher in children with autism, and there were highly significant positive correlations between both BPA (r = 0.400, *p* = 0.004) and 8-OHdG (r = 0.805, *p* = 0.001) with ASD severity. There was a significant positive correlation between body mass index (BMI) and BPA, but the correlation between BMI and 8-OHdG was not significant in children with autism.

A Turkish case-control study by Kondolot et al., 2016 [18] aimed at determining levels of plasma BPA (conjugated plus free form) along with oxidant/antioxidant status in autistic children. BPA and activities of erythrocyte antioxidant enzymes; glutathione peroxidase (GPx1), thioredoxin reductase (TrxR), catalase (CAT), superoxide dismutase (SOD), and glutathione reductase (GR), and levels of erythrocyte antioxidant (GSH) and erythrocyte selenium (Se; co-factor in some antioxidant enzymes), as well as plasma TBARS (no separation step used) and plasma carbonyl levels, were measured in autistic children (classic autism (*n* = 27) and pervasive developmental disorder - not otherwise specified (PDD-NOS) (*n* = 10)) vs. controls (*n* = 35). The mean age was 5.7 ± 2.5 years (males: 80% in study cases and 78% in controls). The group diagnosed PDD-NOS had higher BPA levels than both the control (*p* = 0.003) and classic autism groups (*p* = 0.003), but the classical autism group did not have significantly different BPA levels than controls. Carbonyl levels were significantly higher in the combined group with both classical autism and PDD-NOS) than in controls (*p* = 0.025). Selenium levels and GPx1, SOD, and GR activities were higher (*p* = 0.013; *p* = 0.002; *p* = 0.03; *p* < 0.001, respectively) and CAT activity was lower (*p* < 0.001), in the autistic group compared to controls. However, no associations between BPA concentrations and TBARS or protein carbonyls were found. In addition, no associations between BPA concentrations and erythrocyte GPx1, TrxR, CAT, SOD, or GR activities, or GSH or Se levels, were found.

In a Turkish case-control study by Erden et al., 2014 [19], the relationship between chronic obstructive pulmonary disease (COPD) and BPA, C-reactive protein (CRP), MDA, and total thiol levels (all in serum) was investigated (COPD: *n* = 50; 61.6 ± 11.2 years, 44 males/6 females); controls: *n* = 33; 57.6 ± 11.1 years, 29 males/4 females). In COPD patients, BPA (*p* < 0.001) and CRP (*p* = 0.03) levels were significantly higher than in controls. Total thiol levels (spectrophotometric analysis) were significantly lower (*p* = 0.041) in COPD patients than in controls, whereas MDA levels (no high-performance liquid chromatography (HPLC) separation used before UV analysis) did not differ. Creatinine was not measured. No correlation between BPA and oxidative stress biomarkers was reported, but a non-significant linear relationship (*p* = 0.07) between BPA and CRP was found.

In an Egyptian case-control study by Omran et al., 2018 [20], the association between urinary BPA levels, based on one spot urine sample during the daytime, and infertility-related factors, i.e., semen quality and sperm DNA integrity, was investigated in infertile male patients (*n* = 50) and matched fertile controls (*n* = 50) in Upper Egypt. Semen quality evaluated as sperm concentration, morphology, and motility, and oxidative stress, as total antioxidant activity and MDA levels, were determined along with sperm DNA damage using the alkaline Comet assay. Seminal cell-free plasma was analyzed for total antioxidant activity using an H_2_O_2_ reaction-based kit, and MDA was measured using TBARS (no separation step used). Whereas BPA concentrations were similar (with or without creatinine adjustment), MDA levels were higher (*p* = 0.012) and total antioxidant levels lower (*p* = 0.001) in patients than controls. Overall (*n* = 100), BPA levels were positively correlated with seminal plasma lipid peroxidation (r = 0.298, *p* < 0.01), but slightly negatively correlated with total antioxidant levels (r = −0.004, *p* > 0.05), although this effect was not significant. However, effects were significant for some of the infertile groups subdivided according to the main defects observed that all had negative correlations.

In a South Korean intervention study (single-blinded randomized clinical trial), Yang et al., 2014 [21] studied the intake of Korean red ginseng (KRG) associated with BPA in young non-smoking Korean women with gynecological complaints, mostly menstrual irregularity/pain (KRG: *n* = 11; 22.91 ± 1.81 years); placebo: *n* = 11; 22.73 ± 1.68 years). Total BPA from four morning spot urinary samples taken on day 0, 4, 8, and 14 and MDA (HPLC separation used) were positively associated (slope = 0.88, r^2^ = 0.10, *p* < 0.01). KRG decreased both BPA (*p* < 0.05) and MDA (*p* < 0.05) levels. Creatinine was not measured.

#### 3.2.2. Pregnant Women and Their Fetuses/Newborns

In a nested case-control study, Ferguson et al., 2016 [22] investigated associations between BPA exposure and oxidative stress and inflammation in pregnant women in the USA (*n* = 482). Participants were recruited early in pregnancy and provided urine and plasma at up to four visits (at median 10, 18, 26, and 35 weeks gestation). Total (free + conjugated) BPA, as well as 8-OHdG and 8-isoprostane, were measured in urine at each visit. Inflammation markers, including C-reactive protein (CRP) and four cytokines (interleukin (IL)-1β, IL-6, IL-10, and tumor necrosis factor (TNF)-α) were measured in plasma obtained at the same time points. In adjusted linear mixed models, an interquartile range (IQR) increase in BPA was associated with significant increases, 5% in 8-OHdG (*p* = 0.03) and 9% in 8-isoprostane (*p* = 0.02). In a model adjusted for specific gravity and gestational age at sample collection (*n* = 481 subjects, 1519 observations), BPA was significantly associated with increases in 8-OHdG and 8-isoprostane; % change (95% confidence interval (CI)) with IQR increase in BPA was 8.62% (3.93, 13.5; *p* < 0.01), and 16.2% (8.61, 24.4; *p* < 0.01), respectively. The association for 8-OHdG was similar and the association for 8-isoprostane slightly lower when models were also adjusted for covariates such as race/ethnicity, education level, health insurance provider, and pre-pregnancy BMI (*n* = 460 subjects, 1457 observations). Additionally, they observed significantly higher IL-6 concentrations in association with an IQR increase in BPA (8.95% increase). The authors concluded that these systemic changes consequent to BPA exposure may mediate adverse birth outcomes and/or fetal development.

A Puerto Rican cohort study with repeated measurements of exposure and outcomes during pregnancy by Watkins et al., 2015 [23] investigated the relationship between exposure to phenols and parabens with inflammation and oxidative stress in pregnant women (*n* = 105) aged 18‒40 years at less than 20 weeks gestation. Spot urinary bisphenol measurements were performed as well as urinary biomarkers for oxidative stress (measured three times during pregnancy at 16‒20, 20‒24, and 24‒28 weeks of gestation) in 54 subjects (146 measurements) and plasma markers of inflammation (measured twice during pregnancy at 16‒20 and 24‒28 weeks of gestation) in 105 subjects (187 measurements). Linear mixed models were used to assess potential covariates. An IQR increase in urinary BPA was associated with a 21% higher 8-OHdG (*p* = 0.001) and 29% higher 8-isoprostane (*p* = 0.0002) levels in samples after adjustment for urinary specific gravity, study visit, maternal pre-pregnancy BMI, and maternal education. No associations were found between BPA and any of the markers of inflammation (IL-1β, IL-6, IL-10, TNF-α, or CRP).

In a prospective cohort study with 26‒32 weeks of follow-up by Veiga-Lopez et al., 2015 [24], the impact of BPA on free fatty acids and oxidative stress dynamics was assessed in pregnant women (*n* = 24) living in Michigan, USA. After collection of maternal blood at first trimester (gestational weeks 8–14) and pair-matched umbilical cord blood at term, unconjugated BPA (uBPA) and BPA-glucuronide (BPA-G) were measured in extracted plasma. Thereafter, data were split into low and high uBPA groups (*n* = 12 in each) based on maternal levels. Pregnant mothers with high uBPA levels had higher levels of 3-NO_2_Tyr (*p* < 0.005) compared with those with low uBPA, although the correlation between uBPA and 3-NO_2_Tyr in plasma was not, but nearly, significant (r = 0.398, *p* = 0.054). In mothers, a significant Pearson correlation was found between the related BPA-G and 3-NO_2_Tyr levels (r = 0.440, *p* < 0.05). Cord blood from mothers exposed to higher uBPA levels during gestation had higher 3-NO_2_Tyr levels compared to the low uBPA group (*p* < 0.01). Analysis of samples from both groups, low and high uBPA combined, found a positive correlation between maternal and cord blood 3-NO_2_Tyr levels (r = 0.475, *p* = 0.019). Additionally, 3-ClTyr and diTyr were measured in plasma, but they did not differ between high and low uBPA exposed mothers or in cord blood. Moreover, no correlation was found with 3-ClTyr and diTyr when combining the low and high uBPA groups. In mothers, high BPA was also associated with increased palmitic acid (a free fatty acid) in plasma, and palmitic acid correlated positively with 3-NO_2_Tyr.

In two cross-sectional studies in a pregnancy cohort with 2‒13 weeks of follow-up by Huang et al., 2017 [25] and 2018 [26] on Taiwanese mother/fetus pairs (total *n* = 241 [25] and *n* = 244 [26]), largely the same study participants were included. In mothers with a live singleton pregnancy, BPA and biomarkers of oxidative (8-OHdG) and nitrosative (8-NO_2_Gua) stress and biomarkers of lipid peroxidation (8-isoprostane and HNE-MA) were measured in maternal urine collected in the third trimester (gestational age 27‒38 weeks). Inflammatory markers CRP, IL-6, and TNF-α, and antioxidant enzyme glutathione peroxidase (GPx), were measured in maternal and umbilical cord blood plasma. In adjusted models (oxidative stress biomarkers were normalized to creatinine levels, whereas BPA, antioxidant, and inflammatory biomarkers were not), a significant positive association between third-trimester maternal BPA and 8-isoprostane was found (β = 4.5, *p* = 0.05), but no association was found between BPA and 8-OHdG, 8-NO_2_Gua, or HNE-MA. BPA levels were inversely associated with maternal (β = −30.98, *p* = 0.04) and cord blood (β = −29.40, *p* = 0.01) plasma GPx levels [25]. In the 2018 follow-up study [26], BPA was inversely associated with penis length (β = −4.43 mm, *p* = 0.005) among boys who were born to mothers with high levels of 8-isoprostane.

In a Taiwanese cross-sectional study by Chang et al., 2019 [27] within a pregnancy cohort (the same as by Huang et al., 2017 [25] and 2018 [26] above) with 2‒13 weeks of follow-up, the role of maternal BPA exposure on birth outcomes through oxidative stress was investigated. Urine was collected from pregnant women (*n* = 186; average age 33.3 years) in northern Taiwan in their third trimester (gestational age 27‒38 weeks). Urinary BPA, 8-OHdG, 8-NO_2_Gua, 8-isoprostane, and HNE-MA were measured and levels were adjusted to creatinine. A significant, but weak, correlation was observed between maternal BPA and 8-isoprostane (r = 0.17, *p* = 0.02), whereas no correlation was found between BPA and the other biomarkers of oxidative stress. A non-significant trend between increased BPA with decreased head circumference and birth length was observed. Employing causal mediation analysis using a directed acyclic graph (DAG), a mediated effect of oxidative stress between BPA exposure and birth outcomes, e.g., neonatal head circumference, was not observed.

#### 3.2.3. Children and Adolescents

In a Chinese longitudinal cohort study by Zhou et al., 2019 [28], school children (*n* = 275; 140 boys (50.9%), 135 girls (49.1%)) in East China were investigated for bisphenols and their association with oxidative damage to nucleosides. At first urine sampling, the children were 7‒11 years old and follow-ups were carried out one year apart for the next two years (three measurements in total). Nine BPs (BPA, BPB, BPAF, BPAP, BPE, BPF, BPP, BPS, and BPZ), and 8-OHdG and 8-OHG, as biomarkers of oxidative DNA and RNA damage, respectively, were measured in first-morning urine samples. Concentrations of BPs and oxidative damage biomarkers were creatinine-corrected. Concentrations of 8-OHdG were about 6-fold higher than 8-OHG. A linear mixed model was used for repeated measures analysis. An IQR, the difference between 25th‒75th percentile increase in urinary BPA, was associated with a 12.9% increase in 8-OHdG (*p* < 0.001) and a 19.4% increase in 8-OHG (*p* < 0.001). The children were mainly exposed to BPA, BPS, BPF, and BPAF, and also an IQR increase of their sum was associated with a 17.4% increase in 8-OHdG (*p* < 0.001) and a 25.9% increase in 8-OHG (*p* < 0.001). Moreover, BPS was positively associated with 8-OHG (*p* < 0.006), but not with 8-OHdG.

In a Brazilian cross-sectional study from Rocha et al., 2018 [29], concentrations of 40 endocrine-disrupting chemicals (EDCs) including nine BPs (BPA, BPS, BPAP, BPB, BPP, BPF, BPAF, BPZ, and BPM) and 8-OHdG were investigated in spot urines from 300 urban resident school children (aged 6‒14 years; 149 males, 151 females) from five geographic regions. Among the bisphenols, BPA was detected in 98% of the samples. The other bisphenols were found at much lower detection rates. 8-OHdG was detected in 94.6% of the samples and Spearman rank correlations were found between urinary concentrations of 8-OHdG and BPA (r = 0.261, *p* < 0.01) as well as with eight other EDCs (non-BPs). Clustering of the data based on 14 EDCs to describe exposure, demography (age, gender, geographical location), and 8-OHdG resulted in two distinct clusters of samples, in which 8-OHdG was the most critical parameter that differentiated the two clusters. The results showed that co-exposure to BPA and nine other ECDs (non-BPs) was associated with oxidative DNA damage in the study population.

In a cross-sectional study by Bono et al., 2019 [30], morning urinary sample levels of glucuronic acid of BPA (GlcA-BPA) and 8-isoprostane were measured in healthy school children (*n* = 223; 7‒19 years old (119 males: 53.4%) living in the town of Chivasso, close to Torino in northern Italy. Levels were normalized to creatinine. Piecewise linear robust regression identified a statistically significant association between 8-isoprostane and GlcA-BPA, but only from ≥ 6 ng/mg creatinine (*p* < 0.001).

A cross-sectional study by Lv et al., 2016 [31] investigated whether exposure to BPA was associated with oxidative stress in Chinese children in Guangzhou aged 3‒6 years (*n* = 96; 59 boys, 37 girls). For single spot urinary BPA and 8-OHdG concentrations, a significant association (*p* < 0.05, Spearman rank correlation) in the unit of µg/L was found, but which disappeared after creatinine corrections (unit of µg/g creatinine). A positive association (r = 0.240, *p* = 0.016; multiple linear regression) was found when the µg/L values were transformed to natural logarithms (ln); 1% increase in BPA generated a 0.15% increase in 8-OHdG.

#### 3.2.4. Adults

In a Chinese cross-sectional study with repeated measurements by Wang et al., 2019 [32], spot urine samples were collected from non-smoking men (*n* = 11; 21‒28 years old) over a 3-month period. Participants were asked to collect all urine voids throughout the day and night on days 0, 1, 2, 3, 4, 30, 60, and 90 (529 out of 535 possible spot samples were collected), giving data on spot samples, first morning voids and 24 h samples. Urinary BPA, BPF, BPS, 8-OHdG, 8-isoprostane, and HNE-MA were measured and levels were creatinine-adjusted. The detection rate for BPS was low (13%) and BPS data was not analyzed further. The results showed that the collection of repeated specimens from each individual is recommended since a high degree of within-subject variability was found. Elevated urinary levels of BPA and BPF were associated with increased oxidative stress biomarkers. Urinary BPA was positively associated with 8-OHdG (r = 0.19, *p* < 0.001), HNE-MA (r = 0.10, *p* = 0.03), and 8-isoprostane (r = 0.10, *p* = 0.03), whereas BPF was positively associated with HNE-MA (r = 0.11, *p* = 0.01) and 8-isoprostane (r = 0.10, *p* = 0.02), but negatively (not significantly) associated with 8-OHdG (r = −0.05, *p* = 0.30).

A cross-sectional South Korean study by Hong et al., 2009 [33] investigated whether various chemical exposures caused increased oxidative stress in healthy urban residing adults (*n* = 960; 446 men, 514 women). After adjustment for confounders, e.g., age, sex, weight, smoking, and exercise, urinary 8-OHdG or MDA levels (HPLC separation used) were not associated with BPA levels in single morning urinary samples. No adjustment was done for creatinine.

In a cross-sectional study on South Korean men (*n* = 259), premenopausal (*n* = 92), and postmenopausal (*n* = 134) women, BPA exposure was investigated for induction of oxidative stress and inflammation by Yang et al., 2009 [34]. BPA in single morning urinary samples was significantly positively associated with urinary MDA (β = 0.056‒0.066, *p* = 0.006‒0.008 in three out of three models after adjustment for confounding factors (HPLC separation used), 8-OHdG (β = 0.072‒0.103, *p* = 0.008‒0.025 in 3 of 3 models) and serum CRP (β = 0.113, *p* = 0.029 in 1 of 3 models) in postmenopausal women, but not in men and premenopausal women. Urine concentrations were creatinine-adjusted.

In a cross-sectional Saudi Arabian population study by Asimakopoulos et al., 2016 [35], healthy persons (*n* = 130; both genders, aged 1‒87 years, median age 35) living in Jeddah were investigated for associations between 57 xenobiotics (8 BPs: BPA, BPAF, BPAP, BPS, BPF, BPP, BPZ, and BPB) and oxidative stress in single spot urine samples. After creatinine adjustment of urinary BPs and 8-OHdG concentrations, BPA (r = 0.38, *p* < 0.0001) and BPS (r = 0.30, *p* < 0.0005) were significantly associated with 8-OHdG. In addition, all eight BPs together were positively associated (r = 0.43, *p* < 0.0001) with 8-OHdG.

In a cross-sectional Singaporean study, paired indoor dust and urine samples were collected from healthy participants (*n* = 33; 20 men and 13 women, aged 22‒37 years) by Liu et al., 2019 [36]. BPA, BPS, and various bisphenol A diglycidyl ethers (BADGEs) were measured. Single morning urinary sample levels of BPA and oxidative stress were adjusted for specific gravity. The results showed that 8-OHdG levels in urine samples were positively correlated with urinary BPA levels (r = 0.353, *p* < 0.05) and BMI (r = 0.431, *p* < 0.05), suggesting that elevated oxidative stress might be associated with BPA exposure and obesity.

In a South Korean cross-sectional study by Choi et al., 2019 [37], serum and single spot urine samples were collected from adult Koreans (*n* = 1000; 17‒70 years old, ≈50% of each sex) with the objective of measuring BPA and serum bilirubin (antioxidant). After the exclusion of participants below 20 years of age, serum samples with too low serum volume and urinary samples with too high creatinine concentration, 709 urine and 752 serum samples remained. After analysis, serum samples with too low chemical concentrations were also excluded, as were samples for which several analytical parameters were missing, leaving 585 urinary and 465 serum samples. After enzymatic hydrolysis of conjugated BPA, total concentrations of BPA were measured in urine and serum. Urinary concentrations were adjusted for creatinine. Multivariate regression identified an inverse association between urinary BPA and serum bilirubin levels (β = −0.071, *p* < 0.0001), whereas no association was found between serum BPA and serum bilirubin.

In a South Korean intervention study by Yi et al., 2011 [38], young non-smoking women (*n* = 14; age 24.4 ± 4.0 years) were given wheat sprout juice for 14 days and the effects on BPA levels and oxidative stress were investigated. Total urinary BPA levels, based on first voids of morning urine sampled on days 0, 3, 7, and 14 of intervention, were positively associated with urinary 8-OHdG levels (slope = 1.47, *p* = 0.18 (not significant)), as well as with urinary MDA levels (HPLC separation used) (slope = 0.85, *p* = 0.03). Levels of BPA, but not 8-OHdG and MDA, were creatinine-adjusted. Wheat sprout juice intake lowered urinary BPA levels (*p* = 0.02) as well as 8-OHdG and MDA levels, although not significantly.

#### 3.2.5. Elderly

Two South Korean prospective cohort studies with repeated measurements by the same group, Kim et al., 2016 [39], and Kim and Hong, 2017 [40], investigated the role of genetic polymorphisms in oxidative stress-related genes on BPA exposure in elderly Koreans. Urine samples for BPA measurements were collected during up to five visits from the subjects for two years, out of which many participated in both studies. Blood samples for genotyping were collected at three time points. The repeated measurements were analyzed using GLIMMIX models. In the first study, Kim et al., 2016 [39] investigated the relationship between BPA exposure, liver function, and modification of genetic polymorphisms in oxidative stress-related genes in elderly (≥60 years, mean age 70.6, *n* = 471; 122 (25.9%) men, 349 (74.1%) women). Genomic DNA from human peripheral blood lymphocytes was analyzed for single nucleotide polymorphisms (SNPs) in cyclooxygenase 2 (*COX2* or *PTGS2*), epoxidehydrolase 1 (*EPHX1*), catalase (*CAT*), and superoxide dismutase 2 (*SOD2* or *MnSOD*) genes. Significant associations, as significant odds ratios, of BPA with abnormal liver function were reported in Koreans with certain polymorphisms in genes associated with oxidative stress, such as *COX2*, *CAT*, *EPHX1*, and *SOD2*.

In the second study, Kim and Hong, 2017 [40] further investigated whether BPA exposure induced oxidative stress as MDA in elderly Koreans (≥60 years, mean age 70.8, *n* = 548; 142 (25.9%) men, 406 (74.1%) women) and extended the battery of analyzed oxidative stress-related gene polymorphisms to *COX2*, *EPHX1*, heat shock protein 70-hom (*HSP70-hom*), paraoxonase 1 (*PON1*), endothelial nitric oxide synthase (*eNOS*), *CAT*, dopamine receptor D2 (*DRD2*), *SOD2*, and myeloperoxidase (*MPO*). After adjusting for covariates, but not creatinine, a significant positive association was found between urinary BPA and MDA (HPLC separation used) in both male and female participants (males: β = 0.19 and *p* = 0.0003; females: β = 0.18 and *p* < 0.0001; total: β = 0.18 and *p* < 0.0001) regardless of any genotype of the nine oxidative stress-related genes.

#### 3.2.6. Exposures Based on Residence Area

In a Chinese cross-sectional study by Zhang et al., 2016 [41], it was investigated if exposure to eight different bisphenols (BPA, BPAF, BPAP, BPS, BPF, BPP, BPZ, and BPB) in persons aged 0.4‒87 years (*n* = 116; 66 males, 50 females) living near an e-waste recycling region in Longtang Town, Qingyuan City, China, or in rural (80 km northwest of Longtang, *n* = 22) or urban (Guangzhou, 60 km southeast of Longtang, *n* = 20) reference areas displayed increased oxidative stress. In urinary samples based on first morning voids, BPA, BPS, and BPF were detected with frequencies >90% in persons living near e-waste facilities, with geometric mean concentrations of 3.75, 0.469, and 0.435 µg/g creatinine, whereas these values were 1.78, 1.51, and 1.12 µg/g creatinine in the urban reference area and 1.52, 1.03, and 0.219 µg/g creatinine in the rural reference area, for BPA, BPS, and BPF, respectively. The other five bisphenols were rarely found regardless of sampling location. In the e-waste dismantling location, urinary 8-OHdG was significantly and positively correlated with urinary BPA (r = 0.413, *p* < 0.001) and PBS (r = 0.398, *p* < 0.001), but not with BPF (r = 0.118, *p* = 0.208). Concentrations of bisphenols, but not 8-OHdG, were creatinine-adjusted. Similar correlations between 8-OHdG and BPA and BPS, but not BPF, were also observed when using combined data from the two reference areas.

Only seven papers had looked at associations between bisphenols, oxidative stress biomarkers, and a specific health outcome (Table 1). NAFLD patients had higher plasma and urine BPA and TBARS levels and SOD and CAT activities versus controls [14]. Children with ADHD had higher BPA and 8-OHdG levels vs. controls [16]. Children with ASD had higher BPA and 8-OHdG than controls, and both parameters correlated positively with ASD severity [17]. The other study on autism in children found that the group with a pervasive developmental disorder – not otherwise specified (PDD-NOS), but not the group with classic autism, had higher BPA levels than controls, carbonyl levels were significantly higher in the combined group with both classical autism and PDD-NOS than controls, and selenium levels and GPx1, SOD, and GR activities were higher and CAT activity was lower in the autistic group compared with controls [18]. However, no associations between BPA concentrations and TBARS, protein carbonyls, erythrocyte GPx1, TrxR, CAT, SOD, or GR activities, or GSH or Se levels, were found [18]. COPD patients had higher BPA concentrations in serum, but lower total thiol levels than controls and serum MDA did not differ between the groups [19]. In infertile men, BPA concentrations were similar, MDA levels higher, and total antioxidant levels lower than in controls, and overall BPA levels correlated positively with seminal plasma lipid peroxidation [20]. In young women with gynecological complaints, urinary total BPA and MDA levels were positively associated [21].

Overall, the 27 studies point towards an association between BPA and oxidative stress, based particularly on the 8-OHdG and 8-isoprostane biomarkers (Table 2). Most of the studies on 8-OHdG, mainly measured in urine, only one in serum, found a positive association, although a few studies also found no associations. In addition, the related single study on 8-OHG in urine found a positive association. A few 8-OHdG studies can be interpreted as equivocal (positive, or no association, depending on how the analyses were performed), but none found a negative association. For 8-isoprostane, all five studies (all in urine), with the three Taiwanese pregnancy studies performed on the same cohort counted as one, found a positive correlation. Additionally for MDA, when using a separation step before analysis, more studies reported positive associations than reported no associations. For the other oxidative stress biomarkers, including unspecific MDA analysis and other aldehydes using TBARS, the outcomes were more mixed and studies were too few to conclude. With increased oxidative stress levels, antioxidant enzymes would be expected to increase. Thus, that Huang et al., 2017 [25] found a negative association between GPx and BPA was the only effect association among the papers that went in an opposite direction in terms of oxidative stress. However, the use of antioxidant enzyme levels as biomarkers of oxidative stress is somewhat uncertain since they may also participate in other reactions or be time-dependent. BPA was most often detected and reported, followed by BPF and BPS as the second most frequently measured BPs, although in few studies. Thus, dominantly positive associations, but also some equivocal results, but only one negative association, were found between BPA and oxidative stress biomarkers.

### 3.3. Suggestions of Analytical Methods for the Identified Biomarkers of Oxidative Stress and Evaluation of Their Strengths and Weaknesses

The most frequently measured biomarkers of oxidative stress in human bisphenol studies over the last 12 years were 8-OHdG, 8-isoprostane, and MDA. Our suggestions of suitable analytical methods and strengths and weaknesses of each biomarker are described in Table 3. However, several factors influence choosing the analytical method, e.g., availability of instrumentation and commercial kits, need for derivatization (particularly for small hydrophilic molecules that are poorly retained by the separating column), availability of standards (often isotopically labeled for mass spectrometry), desired specificity and sensitivity, research budget, personal skills, etc. Most of the small molecule biomarkers in Figure 1 can be detected using liquid or gas chromatography-mass spectrometry (LC-MS or GC-MS) and these methods were also the most common choices in the included articles. Tandem MS (i.e., LC-MS/MS and GC-MS/MS) offers extra high sensitivity but is also a large investment.

## 4. Discussion

Overall, the identified studies indicated that exposure to BPA was positively associated with increased urinary levels of 8-OHdG, MDA, and 8-isoprostane. However, there were also some studies that found no associations, or that were equivocal for some of the oxidative stress effect biomarkers. Notably, only one study (on GPx) reported a negative association in terms of effect biomarkers of oxidative stress (note that a negative statistical association between BPA and antioxidants shall be regarded as a positive association in terms of effects on the health of oxidative stress). Since the mentioned oxidative stress biomarkers in serum are ultimately excreted in the urine, measurement in urine is preferred over serum or plasma. Moreover, urine samples can be obtained repeatedly due to their non-invasiveness, offering the advantage of evaluating exposure to bisphenols and oxidative stress biomarkers throughout time.

Analysis of MDA generally relies on reaction with 2-thiobarbituric acid (TBA) under heating (30‒60 min., 70‒100 °C) where artefactual formation of MDA or other aldehydes can take place [42,43]. TBA will also react with other aldehydes (altogether called TBA reactive substances (TBARS), and TBA-MDA products should, therefore, be analyzed using a separation method such as HPLC [42,44], but this is not always the case, e.g., when commercial kits based only on colorimetric detection are used. Therefore, the use of MDA as an oxidative stress biomarker can be questioned as the obtained results are less reliable if a separation step is not included in the analytical procedure. MDA can also be measured using gas chromatography-mass spectrometry (GC-MS) after derivatization under milder conditions [43,45]. Accounting for the unclarity regarding how much artefactual MDA is formed during MDA derivatization, and that some studies have not performed HPLC separation of the formed TBA_2_-MDA complex prior to analysis, urinary 8-OHdG and 8-isoprostane appear to be the most suitable effect biomarkers of oxidative stress.

As a limitation, however, these biomarkers are not specific for BPA, and serum/urinary oxidative stress biomarkers are often not specific to a certain organ. They may also be formed in foods (reacting with proteins and DNA) and not in the body and are therefore unspecific for BPA exposure.

Regarding the other biomarkers, the single study having measured both 8-OHdG and 8-OHG [28] found a stronger association between BPA, or the sum of the four most dominant BPs, and 8-OHG compared with 8-OHdG, despite lower levels of 8-OHG. Urinary HNE-MA has the potential for becoming a reliable biomarker of lipid peroxidation, however, yet there are few studies available. Increased antioxidant enzyme activities of e.g., CAT and SOD, most likely reflect increased ROS levels but are not proof of oxidative damage. Lowered antioxidant levels can result from oxidative stress although some antioxidants, e.g., GSH, also get depleted in enzymatic reactions unrelated to oxidative stress. GSSG or GSSG/GSH in serum is sometimes used as a biomarker for oxidative stress. However, GSH also acts as an electron donor in several biochemical processes, limiting the specificity not only to oxidative stress. Few studies have investigated the relationship between BPA and biomarkers of nitrosative stress. Regarding RNS, which can be seen as a type of ROS, one study found an association between 3-NO_2_Tyr and BPA [24], but more studies are needed to confirm this finding. There seems to be no clear mechanism yet for how bisphenols would lead to increased RNS (e.g., ONOO^−^, NO_2_), but it may be due to increased levels of O_2_^•−^ caused by bisphenols, that subsequently react with NO^•^, forming ONOO^−^. For the other RNS generated biomarkers, no associations with bisphenols were found, but there was only a single study.

Nearly half of the included studies (12 of 27) had a cross-sectional design, which does not allow the assessment of cause and effect relationships because the exposure and the health outcomes are measured in the study participants at the same time. Therefore, a causal association between the exposure to bisphenols and oxidative stress and further to the various health outcomes cannot be established with certainty based on the cross-sectional studies. Many of the oxidative stress biomarkers (Table 3) are not organ-specific, and for this reason, they are also difficult to link to a certain disease. Prospective studies are needed and may also include mediation analyses. The role of oxidative stress as a link between BPA exposure and specific health outcomes can be evaluated using mediation analysis as was done by Chang et al. (2019) using DAG [27]. Broadly, mediation analysis estimates the potential indirect effect exerted by a mediatory variable in the causal path between a given exposure and outcome [46].

Only seven papers had looked at associations between bisphenols, oxidative stress biomarkers, and a specific health outcome (Table 1). One study had a prospective cohort design, five were case-control studies and one study was a single-blind randomized clinical trial. They all found higher levels of BPA and various oxidative stress biomarkers in the patient groups versus the controls group, or a positive correlation between BPA levels and oxidative stress biomarker levels in the patient group. However, since only one, or two studies on autism in children, were found, no firm conclusions can be drawn on the causal link between BPA, oxidative stress, and these health outcomes.

Several of the measured biomarkers of oxidative stress are implicated in adverse outcome pathways (AOPs), mostly as key events (KEs), and rarely linked to a specific adverse outcome (AO) (see Appendix B). Specifically, none of the oxidative stress biomarkers found in these studies were listed in AOPs linked to any of the health outcomes included in our literature search (Appendix A).

In these human studies, exposure is often estimated from measurements of the substances in the urine, often only from one spot urine sample per person and/or time point, and will therefore not take into account the within-person variability in urine concentrations [47]. Non-persistent chemicals such as bisphenols have short physiological half-lives, and thus, a single measurement cannot provide information on long-term exposures. The reported bisphenol exposures in the summarized studies will therefore not necessarily reflect the real-life exposures. Sun et al. [47] concluded that the urinary excretion of various biomarkers such as BPA is reasonably reproducible in 24-h urine samples that are collected within a few days or ≤1 year, and that three 24-h samples are sufficient for the measurement of long-term exposure status in epidemiologic studies. Another paper showed that for non-persistent chemicals, collecting and pooling three samples per day instead of all daily samples, efficiently estimates exposures over a week or more [48]. Urinary BPA levels were in the low μg/L urine range except for one study [21] that reported low ng/L urine levels (likely an error as a figure showed μg/L units). Interestingly, one study [30] found that oxidative stress may first begin at a certain BPA threshold (from BPA-G ≥ 6 ng/mg creatinine).

The various studies differ in whether and how adjustment for urinary dilution was done. Some studies analyzed uncorrected data, e.g., units in µg/L, whereas others normalized the urine concentrations to the concentration of creatinine, a muscle breakdown product, or to specific gravity, the ratio of densities between a urine sample and pure water. No study analyzed urine osmolality. As discussed elsewhere [49], adjustment for urinary dilution is recommended to obtain reliable urinary concentrations of BPA or oxidative stress biomarkers, but there is currently no consensus on the most appropriate method, although some recent studies have concluded that specific gravity is superior to creatinine [49]. Urinary concentrations can be ‘standardized’ or ‘corrected for’ (e.g., µg/g creatinine) or ‘adjusted for’ as a covariate in statistical models (i.e., creatinine set as an independent variable when performing multiple regression analysis; units remain as µg/L) [49]. Both approaches were used in the studies included in this paper. Notably, if X-Y type analysis, e.g., BPA vs. 8-OHdG, is performed on the same urine samples, then the creatinine or specific gravity term often cancels out and adjustment is not needed. For blood analyses, normalizations are normally not performed as the blood volume is relatively constant.

If BPA induces oxidative stress as indicated, the mechanisms involved may be related to the metabolism of BPA, which mainly occurs in hepatocytes. The principal BPA metabolism pathway is direct phase II conjugation leading to a glucuronide [50], but a minor route entails cytochrome P-450 oxidation by hydroxylation to a catechol, followed by further transformation to an *o*-quinone through a postulated bisphenol semiquinone RO^•^ intermediate [5]. The catechol-*o*-quinone couple is capable of redox cycling with the generation of ROS [51,52]. Peroxidase [4,5] and tyrosinase [53] were also shown to oxidize BPA into a bisphenol quinone. In addition to oxidative stress, antioxidant depletion, and effects on antioxidant enzymes, as reported in the papers included in this review, other mechanisms have been suggested regarding BPA’s adverse effects, such as a pro-oxidant effect, mitochondrial dysfunction, alteration in cell signaling pathways, and induction of cell death [3]. BPA has also been shown to have both estrogenic and anti-androgenic effects, as well as potential anti-thyroid effects [54].

Humans are exposed to hundreds, if not thousands, of different xenobiotics simultaneously, therefore, animal studies with controlled exposure, in which single chemicals and higher doses can be studied, could contribute to identify suitable oxidative stress biomarkers from bisphenol exposures, which thereafter can be measured in humans. Although none of the identified biomarkers are considered BPA- or organ-specific, they can be assessed repeatedly and non-invasively in urine and may capture disruptions at different biological levels and could help to understand causal relationships.

## 5. Conclusions

From the last 12 years, 27 publications were identified that had investigated the oxidative stress effect biomarkers in human studies of bisphenols. Of these, BPA had been mostly studied and was most often measured in urine. Of the small molecule biomarkers, urinary measurements of 8-OHdG and MDA were most common, followed by 8-isoprostane and HNE-MA. However, MDA has methodological and analytical issues, wherefore urinary or serum/plasma 8-OHdG, 8-isoprostane, and HNE-MA appear to be the most reliable biomarkers of oxidative stress. There were found dominantly positive associations between BPA and the oxidative stress biomarkers, but also equivocal results, and only one negative association (on the antioxidant enzyme GPx). The finding that a single xenobiotic such as BPA at relatively low exposure levels was predominantly positively associated with increased levels of various biomarkers of oxidative stress is thought-provoking. However, if the literature is not biased with studies reporting mainly positive associations, this may be a reality. None of the biomarkers were specific for any of the bisphenols, and more studies, preferably longitudinal with repeated samples taken and using the best available methodology for measuring the oxidative stress biomarkers, are required to conclude whether they are causally linked to specific health outcomes. However, it is well-documented that increased oxidative stress is associated with increased oxidative damage causing DNA mutations, aging, etc.

## Figures and Tables

**Table 1:** Human studies on bisphenols and biomarkers of oxidative stress during years 2008‒2019.

Study Design (Country)	Population Groups (Numbers of Participants)	Bisphenols (Source)	Oxidative Stress Effect Biomarkers (Source)	Outcomes	Reference
**Diseases and health conditions**					
Prospective cohort (Italy)	NAFLD * (*n* = 60) and controls (*n* = 60) living in Naples, subdivided in NAFL (*n* = 30)) and NASH (*n* = 30), investigated before/after BPA-free diet for 1 month	BPA (plasma and urine)	TBARS, SOD, CAT (all in serum)	The NAFLD patients had higher plasma (*p* < 0.0001) and urine (*p* < 0.0001) BPA levels as well as TBARS levels (*p* < 0.01; no separation step used) compared to controls. The plasma BPA levels were significantly higher in NASH patients than in NAFL patients (*p* = 0.041), independent of the presence of T2DM. SOD and CAT activities were higher in the NAFLD group than in controls (*p* < 0.01). After BPA-free diet, TBARS, SOD, and CAT levels were reduced, but not significantly	Dallio 2017 [14]
Case-control (China)	ADHD (*n* = 215) vs. healthy (*n* = 253) children aged 6‒12 years	BPA (urine)	8-OHdG (urine)	ADHD children had significantly higher urinary BPA (*p* < 0.001) and 8-OHdG (*p* < 0.001) levels vs. controls, and 8-OHdG correlated positively with BPA (r = 0.257, *p* < 0.001)	Li 2018 [16]
Case-control (Egypt)	Children ASD (*n* = 49) vs. matched controls (*n* = 40)	BPA (serum)	8-OHdG (serum)	Both BPA (*p* = 0.025) and 8-OHdG (*p* = 0.0001) were significantly higher in children with ASD, and there were highly significant positive correlations between both BPA (r = 0.400, *p* = 0.004) and 8-OHdG (r = 0.805, *p* = 0.001) with ASD severity	Metwally 2018 [17]
Case-control (Turkey)	Children with classic autism (*n* = 27), PDD-NOS (*n* = 10) and controls (*n* = 35)	BPA (plasma)	TBARS and protein carbonyls (plasma). GPx1, TrxR, CAT, SOD and GR activities, GSH, and Se levels (all in erythrocytes)	The PDD-NOS group had higher BPA levels than both control (*p* = 0.003) and classic autism groups (*p* = 0.003), but the classical autism group was not different from controls. Carbonyl levels were significantly higher in the study group (both classical autism and PDD-NOS) than controls (*p* = 0.025). Selenium levels and GPx1, SOD and GR activities were higher (*p* = 0.013; *p* = 0.002; *p* = 0.03; *p* < 0.001, respectively) and CAT activity was lower (*p* < 0.001) in the autistic group vs. controls. However, no correlations between BPA levels and antioxidant enzymes, antioxidants or oxidative stress effect biomarkers were found	Kondolot 2016 [18]
Case-control (Turkey)	COPD patients (*n* = 50) and controls (*n* = 33)	BPA (serum)	MDA and total thiols (serum)	In the serum of COPD patients, BPA concentrations were significantly higher (*p* < 0.001), and total thiol levels significantly lower (*p* = 0041), than in controls. Serum MDA did not differ between the groups	Erden 2014 [19]
Case-control (Egypt)	Infertile male patients (*n* = 50) vs. matched controls (*n* = 50)	BPA (urine)	Total antioxidant activity and MDA in seminal cell-free plasma	BPA concentrations were similar, MDA levels higher (*p* = 0.012) and total antioxidant levels lower (*p* = 0.001), in patients than controls. Overall (*n* = 100), BPA levels were positively correlated with seminal plasma lipid peroxidation (r = 0.298, *p* < 0.01)	Omran 2018 [20]
Intervention (single-blind randomized clinical trial) (South Korea)	Young women investigated for gynecological complaints given KRG (*n* = 11) or placebo (*n* = 11)	BPA (urine)	MDA (urine)	Urinary total BPA and MDA levels were positively associated (slope = 0.88, r^2^ = 0.10, *p* < 0.01). Intervention with KRG decreased both BPA (*p* < 0.05) and MDA (*p* < 0.05) levels	Yang 2014 [21]
**Pregnant women and their fetuses/newborns**					
Nested case-control with repeated measurements of exposure and outcomes during pregnancy (USA)	Pregnant women (*n* = 482), CRP, IL-1β, IL-6, IL-10 and TNF-α measured up to four times in plasma	BPA (urine)	8-OHdG and 8-isoprostane (both urine), up to four measurements	In adjusted models, an IQR increase in BPA was associated with significant increases in oxidative stress biomarkers, 5% in 8-OHdG (*p* = 0.03) and 9% in 8-isoprostane (*p* = 0.02). Significantly higher IL-6 concentrations were associated with an IQR 8.95% increase in BPA	Ferguson 2016 [22]
Cohort with repeated measurements of exposure and outcomes during pregnancy (Puerto Rico)	Pregnant women (*n* = 106, of which 54 had oxidative stress biomarkers analyzed)	BPA (urine)	8-OHdG and 8-isoprostane (both urine), three measurements	An IQR range increase in BPA was associated with 29% higher 8-isoprostane (*p* = 0.0002) and 21% higher 8-OHdG (*p* = 0.001) levels	Watkins 2015 [23]
Prospective pregnancy cohort (USA)	Pregnant women (*n* = 24), plasma and their umbilical cord at birth	uBPA and BPA-G (blood plasma)	3-NO_2_Tyr, 3-ClTyr, and diTyr (all in plasma)	A positive association between BPA (as BPA-G: r = 0.440, *p* < 0.05; as uBPA: r = 0.398, *p* = 0.054 (not significant)) and 3-NO_2_Tyr levels (but not 3-ClTyr and diTyr) was found in plasma from pregnant mothers. In cord blood, 3-NO_2_Tyr levels were higher (*p* < 0.10) when mothers belonged to the highest uBPA exposed half	Veiga-Lopez 2015 [24]
Cross-sectional within a pregnancy cohort (Taiwan)	Study on mother/fetus pairs (*n* = 241 (244 in follow-up study)	BPA (urine)	8-OHdG, 8-NO_2_Gua, 8-isoprostane and HNE-MA levels in 3rd trimester maternal urine. GPx in maternal and umbilical cord blood plasma	Positive associations between maternal BPA and 8-isoprostane levels (β = 4.5, *p* = 0.05), but no associations between BPA and 8-OHdG, HNE-MA or 8-NO_2_Gua levels [28,29]. BPA concentrations were inversely associated with maternal (β = −30.98, *p* = 0.04) and cord blood (β = −29.40, *p* = 0.01) plasma GPx levels. BPA was inversely associated with penis length (β = −4.43 mm, *p* = 0.005) among boys who were born to mothers high in 8-isoprostane [29]	Huang 2017 [25] and 2018 [26]
Cross-sectional within a pregnancy cohort (Taiwan)	Pregnant women (*n* = 186) in northern Taiwan and birth outcomes after delivery	BPA (urine)	8-OHdG, 8-NO_2_Gua, 8-isoprostane, and HNE-MA (all urinary)	A significant but weak correlation was observed between maternal BPA and 8-isoprostane (r = 0.17, *p* = 0.02), whereas no correlation was found between BPA and the other oxidative stress biomarkers	Chang 2019 [27]
**Children and adolescents**					
Longitudinal cohort with repeated measurements (China)	School children (*n* = 275), age 7‒11 years at first sampling	BPA, BPB, BPAF, BPAP, BPE, BPF, BPP, BPS, BPZ (all in urine)	8-OHdG and 8-OHG (both in urine)	An IQR increase in BPA was associated with 12.9% (*p* < 0.001) increase in 8-OHdG and 19.4% (*p* < 0.001) increase in 8-OHG. An IQR increase in the sum of the dominant BPs (BPA, BPS, BPF, and BPAF) was associated with 17.4% (*p* < 0.001) increase in 8-OHdG and 25.9% (*p* < 0.001) increase in 8-OHG, respectively. BPS was positively associated with 8-OHG (*p* < 0.006), but not with 8-OHdG	Zhou 2019 [28]
Cross-sectional (Brazil)	Urban resident children (*n* = 300), 6‒14 years, from five geographic regions in Brazil	BPA, BPS, BPAP, BPB, BPP, BPF, BPAF, BPZ, and BPM (all in urine)	8-OHdG (urine)	Significant association between urinary 8-OHdG and BPA (r = 0.261, *p* < 0.01) and a number of other non-BPs EDCs. Other bisphenols had low detection frequency. Co-exposure to 10 EDCs, including BPA, was associated with oxidative DNA damage	Rocha 2018 [29]
Cross-sectional (Italy)	School children aged 7‒19 years (*n* = 223)	GlcA-BPA (urine)	8-isoprostane (urine)	A significant association between 8-isoprostane and GlcA-BPA was found, but only from ≥6 ng/mg creatinine (*p* < 0.001)	Bono 2019 [30]
Cross-sectional (China)	Children aged 3‒6 years (*n* = 96)	BPA (urine)	8-OHdG (urine)	A significant positive association was found (r = 0.240, *p* = 0.016) between BPA and 8-OHdG levels after ln-transformation of unadjusted data	Lv 2016 [31]
**Adults**					
Cross-sectional (with repeated measurements) (China)	Healthy Chinese men (*n* = 11), urine measurements taken repeatedly over 3 months	BPA, BPF, BPS (all in urine)	8-OHdG, 8-isoprostane, and HNE-MA (all in urine)	BPA was positively associated with 8-OHdG (r = 0.19, *p* < 0.001), HNE-MA (r = 0.10, *p* = 0.03), and 8-isoprostane (r = 0.10, *p* = 0.03), whereas BPF was positively associated with HNE-MA (r = 0.11, *p* = 0.01) and 8-isoprostane (r = 0.10, *p* = 0.02), but negatively (not significantly) associated with 8-OHdG (r = −0.05, *p* = 0.30)	Wang 2019 [32]
Cross-sectional (South Korea)	Urban residing adults (*n* = 960)	BPA (urine)	8-OHdG and MDA (both in urine)	8-OHdG and MDA levels were not significantly associated with BPA in Korean urban residing adults	Hong 2009 [33]
Cross-sectional (South Korea)	Adult men (*n* = 259), pre- (*n* = 92) and postmenopausal (*n* = 134) women	BPA (urine)	8-OHdG and MDA (urine)	BPA was positively associated with MDA (β = 0.056‒0.066, *p* = 0.006‒0.008 in 3 of 3 models) and 8-OHdG (β = 0.072‒0.103, *p* = 0.008‒0.025 in 3 of 3 models) in postmenopausal women, but not in men and premenopausal women	Yang 2009 [34]
Cross-sectional (Saudi Arabia)	Healthy persons (*n* = 130), aged 1‒87 years	BPA, BPAF, BPAP, BPS, BPF, BPP, BPZ, and BPB (all in urine)	8-OHdG (urine)	BPA (r = 0.38, *p* < 0.0001) and BPS (r = 0.30, *p* < 0.0005) were significantly associated with 8-OHdG levels. All 8 BPs together were positively associated (r = 0.43, *p* < 0.0001) with 8-OHdG levels	Asimakopoulos 2016 [35]
Cross-sectional (Singapore)	Healthy participants (*n* = 33), 22‒37 years	BPA, BPS, several BADGEs (urine)	8-OHdG (urine)	8-OHdG levels were positively correlated with BPA levels (r = 0.353, *p* < 0.05)	Liu 2019 [36]
Cross-sectional (South Korea)	General population (*n* = 585 persons for urine, *n* = 465 for serum, aged 20‒70 years)	BPA (serum and urine)	bilirubin (serum)	An inverse association between urinary BPA and serum bilirubin levels (β = −0.071, *p* < 0.0001) was found, whereas no association was found between serum BPA and serum bilirubin	Choi 2019 [37]
Intervention (South Korea)	Young women (*n* = 14) given wheat sprout juice for 14 days	BPA (urine)	8-OHdG and MDA (urine)	BPA concentrations were positively associated with MDA levels (slope = 0.85, *p* = 0.03) and 8-OHdG (slope = 1.47, *p* = 0.18 (not significant))	Yi 2011 [38]
**Elderly**					
Prospective cohort with repeated measurements (South Korea)	Elderly (*n* = 471) investigated for liver function and polymorphisms in oxidative stress-related genes	BPA (urine)	SNPs in *COX2*, *EPHX1*, *CAT* and *SOD2* genes in blood cells	Significant associations of BPA with abnormal liver function were found in Koreans with certain polymorphisms in genes associated with oxidative stress, such as *COX2*, *CAT*, *EPHX1*, and *SOD2*	Kim 2016 [39]
Prospective cohort with repeated measurements (South Korea)	Elderly (*n* = 548) investigated for polymorphisms in oxidative stress-related genes and oxidative stress	BPA (urine)	SNPs in *COX2*, *EPHX1*, *CAT*, *SOD2*, *HSP70-hom*, *PON1*, *eNOS*, *DRD2*, and *MPO* genes and MDA (urine)	A significant positive association was found between urinary BPA and MDA in both sexes (males: β = 0.19 and *p* = 0.0003, females: β = 0.18 and *p* < 0.0001) regardless of any genotype of the nine oxidative stress-related genes	Kim and Hong 2017 [40]
**Exposures based on residence area**					
Cross-sectional (China)	Residents (*n* = 116), 0.4‒87 years, living in the e-waste recycling region Longtang Town, Qingyuan City, or in rural or urban reference areas	BPA, BPAF, BPAP, BPS, BPF, BPP, BPZ, and BPB (all in urine)	8-OHdG (urine)	In the e-waste dismantling location, urinary BPA (r = 0.413, *p* < 0.001) and BPS (r = 0.386, *p* < 0.001), but not BPF (r = 0.118, *p* = 0.208), were positively associated with urinary 8-OHdG levels. Similar correlations were also observed for the combined data from the two reference areas	Zhang 2016 [41]

* Abbreviations: attention deficit hyperactivity disorder (ADHD), autism spectrum disorders (ASD), bisphenol A diglycidyl ethers (BADGEs), bisphenol A (4,4′-isopropylidenediphenol) (BPA), bisphenol AF (4,4′-(hexafluoroisopropylidene)-diphenol) (BPAF), BPA glucuronide (BPA-G), bisphenol AP (4,4′-(1-phenylethylidene)bisphenol) (BPAP), bisphenol B (2,2-Bis(4-hydroxyphenyl)butane) (BPB), bisphenol E (1,1-bis(4-hydroxyphenyl)ethane) (BPE), bisphenol F (4,4′-dihydroxydiphenylmethane) (BPF), bisphenol M (4,4′-(1,3-phenylenediisopropylidene)bisphenol) (BPM), bisphenol P (4,4′-(1,4-phenylenediisopropylidene)bisphenol) (BPP), bisphenol S (4,4′-sulfonyldiphenol) (BPS), bisphenol Z (4,4′-cyclohexylidenebisphenol) (BPZ), catalase (CAT), catalase gene (*CAT*), 3-chloro-tyrosine (3-ClTyr), chronic obstructive pulmonary disease (COPD), cyclooxygenase 2 gene (*COX2*), C-reactive protein (CRP), *o*,*o*’-di-tyrosine (diTyr), deoxyribonucleic acid (DNA), dopamine receptor D2 gene gene (*DRD2*), endocrine-disrupting chemicals (EDCs), endothelial nitric oxide synthase gene (*eNOS*), epoxidehydrolase 1 gene (*EPHX1*)glucuronic acid of BPA (GlcA-BPA), glutathione peroxidase (GPx, GPx1), glutathione reductase (GR), glutathione (GSH), 4-hydroxy-2-nonenal-mercapturic acid (HNE-MA), heat shock protein 70-hom gene (*HSP70-hom*), interleukin 1β (IL-1β), interleukin-6 (IL-6), interleukin-10 (IL-10), interquartile range (IQR), 8-iso-prostaglandin F2α (8-isoprostane), Korean red ginseng (KRG), malondialdehyde (MDA), myeloperoxidase gene (*MPO*), non-alcoholic fatty liver disease (NAFLD), non-alcoholic fatty liver (NAFL), non-alcoholic steatohepatitis (NASH), 8-nitro-guanine (8-NO_2_Gua), 3-nitro-tyrosine (3-NO_2_Tyr), 8-oxo-7,8-dihydro-2′-deoxyguanosine (8-OHdG), 8-oxo-7,8-dihydroguanosine (8-OHG), pervasive developmental disorder—not otherwise specified (PDD-NOS), paraoxonase 1 gene (*PON1*), selenium (Se), single nucleotide polymorphisms (SNPs), superoxide dismutase (SOD), superoxide dismutase 2 gene (*SOD2*), 2-thiobarbituric (TBA) reactive substances (TBARS), tumor necrosis factor α (TNF-α), thioredoxin reductase (TrxR), unconjugated BPA (uBPA).

**Table 2:** Summary of positive, equivocal, and negative statistical associations between BPA and oxidative stress biomarkers in human studies.

POSITIVE	NO ASSOCIATION	NEGATIVE ^1^
**8-OHdG ^2^**		
Li 2018 (urine) ^3^ Metwally 2018 (serum) Zhou 2019 (urine) Ferguson 2016 (urine) Watkins 2015 (urine) Yang 2009 (urine; only in postmenopausal women) Wang 2019 (urine) Asimakopoulos 2016 (urine) Liu 2019 (urine) Rocha 2018 (urine) Zhang 2016 (urine)	Huang 2017 and 2018, and Chang 2019 (urine) Lv 2016 (urine; POS in µg/L when unadjusted, but not as µg/L after creatinine normalization, POS as ln of µg/L) Hong 2009 (urine)	-
**8-isoprostane**		
Ferguson 2016 (urine) Watkins 2015 (urine) Huang 2017 and 2018, and Chang 2019 (urine) Bono 2019 (urine; POS at ≥6 ng/mL creatinine) Wang 2019 (urine)	-	-
**MDA (chromatographic separation)**		
Yang 2014 (urine) Yang 2009 (urine; only in postmenopausal women) Yi 2011 (urine) Kim and Hong 2017 (urine)	Hong 2009 (urine)	
**MDA (unspecific detection) and other aldehydes using TBARS**		
Dallio 2018 (serum TBARS) Omran 2018 (seminal plasma)	Kondolot 2016 (plasma TBARS) Erden 2014 (serum)	-
**HNE-MA**		
Wang 2019 (urine)	Huang 2017 and 2018, and Chang 2019 (urine)	-
**8-NO_2_Gua**		
-	Huang 2017 and 2018, and Chang 2019 (urine)	-
**Antioxidant enzymes**		
Dallio 2018 (serum SOD and CAT)	Kondolot 2016 (erythrocytes)	Huang 2017 (maternal plasma and cord blood (GPx)
**Total thiols and GSH**		
-	Kondolot 2016 (erythrocytes)	-
-	Erden 2014 (serum)	-
**Oxidative stress genetic polymorphism (DNA)**		
Kim 2016 (association BPA vs. liver function for certain oxidative stress genes)	-	-
**3-NO_2_Tyr**		
Veiga-Lopez 2015 (plasma)	-	-
**3-ClTyr (plasma) and diTyr**		
-	Veiga-Lopez 2015 (plasma)	-
**8-OHG**		
Zhou 2019 (urine)	-	-
**Bilirubin (serum)**		
-	Choi 2019 (serum)	Choi 2019 (urine)
**Protein carbonyls**		
-	Kondolot 2016 (plasma)	-
**Total antioxidant activity** (no significant outcomes)		
-	-	-

^1^ For antioxidants (consumed during oxidative stress), but not antioxidant enzymes, a negative statistical association with BPA is regarded as positive in terms of effects on health due to oxidative stress. ^2^ Abbreviations: bisphenol A (4,4′-isopropylidenediphenol) (BPA), catalase (CAT), 3-chloro-tyrosine (3-ClTyr), *o*,*o*’-di-tyrosine (diTyr), deoxyribonucleic acid (DNA), glutathione peroxidase (GPx), glutathione (GSH), 4-hydroxy-2-nonenal-mercapturic acid (HNE-MA), 8-iso-prostaglandin F2α (8-isoprostane), malondialdehyde (MDA), 8-nitro-guanine (8-NO_2_Gua), 3-nitro-tyrosine (3-NO_2_Tyr), 8-oxo-7,8-dihydro-2′-deoxyguanosine (8-OHdG), 8-oxo-7,8-dihydroguanosine (8-OHG), positive (POS), superoxide dismutase (SOD), 2-thiobarbituric (TBA) reactive substances (TBARS). ^3^ Source of the oxidative stress biomarker (often the same as for BPA, see Table 1).

**Table 3:** Evaluation of analytical methods and strengths and weaknesses of the identified biomarkers of oxidative stress in human studies on BPA.

Biomarker (Source)	Number of Human Studies ^1^	Associations between BPA and Oxidative Stress Biomarkers	Suitable Analytical Methods	Strengths	Weaknesses
8-OHdG ^2^ (urine)	15	Dominantly positive associations, but also equivocal results. No negative associations	Best options: LC-MS or HPLC-EC. Other options: ELISA, GC-MS	Easy to measure	Not BPA- or organ-specific. May originate from food or endogenous sources. Can display considerable inter- and intra-day variations
8-isoprostane (urine)	5	Only positive associations	Best options: LC-MS or GC-MS	8-isoprostane is specific for lipid peroxidation	Not BPA- or organ-specific
MDA (chromatographic separation) (urine)	5	Dominantly positive associations, but also equivocal results. No negative associations	Best option: derivatization followed by GC-MS or HPLC separation prior to UV or fluorescence analysis	MDA is specific for lipid peroxidation	Risk of artefactual generation during preparatory steps involving excessive heating prior to analysis. Not BPA- or organ-specific
MDA (unspecific detection) and other aldehydes using TBARS (serum, plasma)	4	Two positive, two with no associations	Not recommended: UV or fluorescence analysis of formed chromophore, but no separatory step used in the analysis	May be related to oxidative stress	Unclear what is measured. Risk of artefactual generation during preparatory steps. Not BPA- or organ-specific
Antioxidant enzymes (erythrocytes, plasma, serum)	3	Heterogeneous outcomes	Best option: enzymatic activity assay. Others: Western blot or ELISA	Antioxidant enzymes may be upregulated during conditions of oxidative stress	Not BPA- or organ-specific. High oxidative stress levels may be required to induce additional synthesis of antioxidant enzymes
HNE-MA (urine)	2	One positive, one with no association	Best options: LC-MS, ELISA	HNE-MA is specific for lipid peroxidation	Not BPA- or organ-specific
Total thiols and GSH (erythrocytes, serum)	2	No associations	Best option: LC-MS. Others: kinetic assay or by spectrophotometry	Lowered SH groups may be related to increased oxidative stress	Not BPA- or organ-specific. GSH is used in a broad range of biochemical reactions making it less specific
Polymorphisms in oxidative stress genes (DNA)	2 (1 for liver function, 1 for MDA using separation)	Both studies found positive associations	Best option: DNA gene sequencing using PCR	Poly-morphisms in oxidative stress genes may be related to susceptibility to toxic substances and diseases	Not BPA- or organ-specific. Protein levels may not always reflect the number of gene copies. Proteins can have several functions
3-NO_2_Tyr/3-ClTyr/diTyr (plasma)	1	Positive association for 3-NO_2_Tyr. No association for 3-ClTyr or diTyr	Best option: LC-MS	3-NO_2_Tyr, 3-ClTyr and diTyr are specific for RNS, HOCl and ROS, respectively	Not BPA- or organ-specific
8-OHG	1	Positive association	Best options: LC-MS or HPLC-EC. Others: ELISA, GC-MS	Easy to measure	Not BPA- or organ-specific. May originate from food or endogenous sources. Can display considerable inter- and intra-day variations
8-NO_2_Gua (urine)	1	No association	Best option: LC-MS	8-NO_2_Gua is specific for RNS	Not BPA- or organ-specific
Protein carbonyls (plasma)	1	No association	Best option: ELISA	Protein carbonyls are specific for oxidative stress and are considered stable being only slowly repaired/turned over	Not BPA- or organ-specific. Origin somewhat unclear
Bilirubin (serum and urine)	1	Inverse association (urine) and no association (serum)	Best option: LC-MS	Easy to measure	Not BPA- or organ-specific
Total antioxidant activity (seminal plasma)	1	No association	Best option: commercial H_2_O_2_-based kit	Easy to measure	Not BPA- or organ-specific. Unclear what is measured

^1^ The three Taiwanese studies on the same pregnancy cohort [25,26,27] had recruited subjects during a similar time period and observed largely the same outcomes regarding 8-isoprostane, 8-OHdG, 8-NO_2_Gua, and HNE-MA, and are therefore counted as one study; ^2^ Abbreviations: bisphenol A (4,4′-isopropylidenediphenol) (BPA), (3-chloro-tyrosine (3-ClTyr), *o*,*o*’-di-tyrosine (diTyr), deoxyribonucleic acid (DNA), enzyme-linked immunosorbent assay (ELISA), gas chromatography-mass spectrometry (GC-MS), glutathione (GSH), 4-hydroxy-2-nonenal-mercapturic acid (HNE-MA), hydrogen peroxide (H_2_O_2_), hypochlorous acid (HOCl), high-performance liquid chromatography with electrochemical detection (HPLC-EC), 8-iso-prostaglandin F2α (8-isoprostane), liquid chromatography-mass spectrometry (LC-MS), malondialdehyde (MDA), 8-nitro-guanine (8-NO_2_Gua), 3-nitro-tyrosine (3-NO_2_Tyr), 8-oxo-7,8-dihydro-2′-deoxyguanosine (8-OHdG), 8-oxo-7,8-dihydroguanosine (8-OHG), polymerase chain reaction (PCR), reactive nitrogen species (RNS), reactive oxygen species (ROS), sulfhydryl (SH), 2-thiobarbituric (TBA) reactive substances (TBARS), ultraviolet (UV).

## Appendix A

**Table A1:** Used in the two Pubmed database searches performed at the turn of the year 2017–2018 (limited to years 2008–2017) and on 3 January, 2020, for the years 2018–2019.

Health Endpoint	Bisphenol AND (MeSH Terms OR synonym)
Behavior, neurodevelopment, and neurodegeneration	(Bisphenol) AND (((((((((((((“Behavior”[Mesh]) OR (“Behavior and Behavior Mechanisms”[Mesh] OR “Reproductive Behavior”[Mesh])) OR “Social Behavior Disorders”[Mesh]) OR (“Child Behavior Disorders”[Mesh] OR “Adolescent Behavior”[Mesh])) OR “Antisocial Personality Disorder”[Mesh]) OR (“Infant Behavior”[Mesh] OR “Spatial Behavior”[Mesh])) OR “Sucking Behavior”[Mesh]) OR (“Sexual Behavior, Animal”[Mesh] OR “Sexual Behavior”[Mesh])) OR (“Paternal Behavior”[Mesh] OR “Maternal Behavior”[Mesh] OR “Impulsive Behavior”[Mesh] OR “Feeding Behavior”[Mesh] OR “Exploratory Behavior”[Mesh])) OR (“Compulsive Behavior”[Mesh] OR “Child Behavior”[Mesh] OR “Behavior, Animal”[Mesh])) OR “Mental Disorders”[Mesh])) OR (Behavior OR Neurobehavior OR Neurodevelopment OR Neurology OR Parkinson OR Alzheimer OR Autism OR Hyperactivity OR ASD OR ADHD OR mental retardation OR IQ loss OR internalizing OR externalizing))
Cancer	(Bisphenol) AND (((((((“Neoplasms”[Mesh] OR “Uterine Cervical Neoplasms”[Mesh] OR “Urologic Neoplasms”[Mesh] OR “Liver Neoplasms”[Mesh] OR “Hereditary Breast and Ovarian Cancer Syndrome”[Mesh] OR “Early Detection of Cancer”[Mesh]) OR (“Urogenital Neoplasms”[Mesh] OR “Testicular Neoplasms”[Mesh] OR “Endometrial Neoplasms”[Mesh] OR “Vaginal Neoplasms”[Mesh] OR “Uterine Neoplasms”[Mesh])) OR (“Prostatic Neoplasms”[Mesh] OR “Ovarian Neoplasms”[Mesh] OR “Endocrine Gland Neoplasms”[Mesh])) OR (“Breast Neoplasms”[Mesh] OR “Neoplasms, Germ Cell and Embryonal”[Mesh] OR “Tumor Microenvironment”[Mesh])) OR (“Thyroid Neoplasms”[Mesh] OR “Pituitary Neoplasms”[Mesh] OR “Brain Neoplasms”[Mesh]))) OR (Cancer OR hormone-dependent cancer OR neoplasm OR malignant tumor OR tumor OR tumour) OR (Colon neoplasms))
Endocrine diseases	(Bisphenol) AND (“Endocrine System”[Mesh] OR “Endocrine Glands”[Mesh] OR “Endocrine System Diseases”[Mesh] OR “Hormones”[Mesh] OR “Gonadal Hormones”[Mesh] OR “Placental Hormones”[Mesh] OR “Pituitary Hormones”[Mesh] OR “Growth Hormone”[Mesh] OR “Thyroid Hormones”[Mesh] OR “Gastrointestinal Hormones”[Mesh] OR “Sex Hormone-Binding Globulin”[Mesh] OR “Adrenocorticotropic Hormone”[Mesh] OR “Adrenal Cortex Hormones”[Mesh] OR Endocrine system OR hypothyroidism OR hyperthyroidism OR adrenal)
Immune system and allergy	(bisphenol) AND (“Allergy and Immunology”[Mesh] OR “Hypersensitivity”[Mesh] OR “Rhinitis, Allergic, Seasonal”[Mesh] OR “Food Hypersensitivity”[Mesh] OR “Drug Hypersensitivity”[Mesh] OR “Shellfish Hypersensitivity”[Mesh] OR Allergy OR Hypersensitive OR respiratory allergy OR gastrointestinal allergy OR multiple chemical sensitivity OR allergic hypersensitivity disease OR contact allergy OR “Immune System”[Mesh] OR “Immune System Diseases”[Mesh] OR Immune system OR autoimmune disease OR cytokines OR white cells OR innate immune system OR adaptive immune system)
Obesity, metabolic disorders and cardiovascular diseases	(Bisphenol) AND (“Metabolic Syndrome”[Mesh] OR “Nutritional and Metabolic Diseases”[Mesh] OR “Metabolic Diseases”[Mesh] OR “Metabolism”[Mesh] OR “Glucose Metabolism Disorders”[Mesh] OR “Acidosis”[Mesh] OR “Metabolome”[Mesh] OR “Metabolomics”[Mesh] OR “Receptor, Insulin”[Mesh] OR “Lipolysis”[Mesh] OR “Gout”[Mesh] OR “Diabetes Mellitus, Type 2”[Mesh] OR “Acidosis, Renal Tubular”[Mesh] OR “Homocysteinemia” [Supplementary Concept] OR “Peroxisome Proliferator-Activated Receptor Gamma Coactivator 1-alpha”[Mesh] OR “Obesity”[Mesh] OR “Pediatric Obesity”[Mesh] OR “Obesity, Abdominal”[Mesh] OR “Abdominal obesity metabolic syndrome” [Supplementary Concept] OR “Cardiovascular System”[Mesh] OR “Cardiovascular Abnormalities”[Mesh] OR “Pregnancy Complications, Cardiovascular”[Mesh] OR “Cardiovascular Diseases”[Mesh] OR “Myocardial Infarction”[Mesh] OR obesity OR abdominal obesity OR waist hip ratio OR adipose tissue OR adypokine OR visceral fat OR body fat OR overweight OR Metabolic OR Metabolic disorder OR Metabolic syndrome OR glucose homeostasis OR Hyperlipidemia OR Dyslipidemia OR hypertriglyceridemia OR HOMA-IR OR insulin resistance OR pancreas OR liver OR kidney)
Reproductive diseases	(Bisphenol) AND (reproductive OR puberty OR pregnancy OR infertility OR semen quality OR placenta OR anogenital distance OR hypospadia OR cryptorchidism OR “Reproductive Health”[Mesh] OR “Reproductive Medicine”[Mesh] OR “Reproduction”[Mesh] OR “Reproductive Techniques, Assisted”[Mesh] OR “Infertility”[Mesh])

On 27 January, 2020, a complementary PubMed search using the broad search terms “bisphenol AND oxidative stress, AND biomarker” was performed.

## Appendix B

### Appendix B.1. Adverse Outcome Pathways (AOPs) and Key Events (KEs) Involving BPA or the Biomarkers of Oxidative Stress Reported in the Human Studies (Accessed 15 April 2020)

**BPA** is listed as stressor 521 in AOPWiki, link: https://aopwiki.org/stressors/521. The only AOP listed with BPA as a stressor is AOP 314: Activation of estrogen receptor in immune cells leading to exacerbation of systemic lupus erythematosus (status: under development, included in OECD Work Plan), link: https://aopwiki.org/aops/314.

**Oxidative stress** is listed as KE 1392, in AOP 17: Binding of electrophilic chemicals to SH (thiol)-group of proteins and/or to seleno-proteins involved in protection against oxidative stress during brain development leads to impairment of learning and memory (status: under development, included in OECD Work Plan), link: https://aopwiki.org/aops/17, AOP 220: Cyp2E1 activation leading to liver cancer (status: under review, included in OECD Work Plan), link: https://aopwiki.org/aops/220 and AOP 284: Binding of electrophilic chemicals to SH (thiol)-group of proteins and/or to seleno-proteins involved in protection against oxidative stress leads to chronic kidney disease (status: under development), link: https://aopwiki.org/aops/284.

**ROS** is mentioned in 26 AOPs (3, 17, 27, 37, 38, 41, 54, 144, 148, 149, 173, 207, 213, 216, 220, 238, 257, 260, 272, 277, 282, 293, 294, 296, 298, 311), link: https://aopwiki.org/aops?utf8=%E2%9C%93&search=reactive+oxygen+species+&commit=Search&find_by_id= and in 2 KEs, link: https://aopwiki.org/events?utf8=%E2%9C%93&search=reactive+oxygen+species+&commit=Search&find_by_id=.

**RNS** is mentioned in 7 AOPs (37, 149, 173, 277, 293, 294, 296), link: https://aopwiki.org/aops?utf8=%E2%9C%93&search=reactive+nitrogen+species+&commit=Search&find_by_id= and in 8 KEs, link: https://aopwiki.org/events?utf8=%E2%9C%93&search=reactive+nitrogen+species+&commit=Search&find_by_id=.

**8-OHdG** is mentioned in AOP 17: Binding of electrophilic chemicals to SH (thiol)-group of proteins and/or to seleno-proteins involved in protection against oxidative stress during brain development leads to impairment of learning and memory (status: under development, included in OECD Work Plan), link: https://aopwiki.org/aops/17 and in AOP 296: Oxidative DNA damage leading to chromosomal aberrations and mutations (status: under development), link: https://aopwiki.org/aops/296. It is also listed in KE 1634, link: https://aopwiki.org/events?utf8=%E2%9C%93&search=8-OHdG&commit=Search&find_by_id=.

**8-isoprostane** is mentioned in KE 1498, link: https://aopwiki.org/events?utf8=%E2%9C%93&search=8-isoprostane&commit=Search&find_by_id=.

**MDA** is mentioned in AOP 220: Chronic Cyp2E1 activation leading to liver cancer (status: under review, included in OECD Work Plan), link: https://aopwiki.org/aops/220#stressors and AOP 260: CYP2E1 activation and formation of protein adducts leading to neurodegeneration (status: under development), link: https://aopwiki.org/aops/260. MDA is also listed in 3 KEs, link: https://aopwiki.org/events?utf8=%E2%9C%93&search=malondialdehyde&commit=Search&find_by_id=.

**TBARS/TBA** is mentioned in AOP 17: Binding of electrophilic chemicals to SH (thiol)-group of proteins and/or to seleno-proteins involved in protection against oxidative stress during brain development leads to impairment of learning and memory (status: under development, included in OECD Work Plan), link: https://aopwiki.org/aops/17, AOP 220: Chronic Cyp2E1 activation leading to liver cancer (status: under review, included in OECD Work Plan), link: https://aopwiki.org/aops/220#stressors and AOP 260: CYP2E1 activation and formation of protein adducts leading to neurodegeneration (status: under development), link: https://aopwiki.org/aops/260. TBARS is also listed in 4 KEs, links: https://aopwiki.org/events?utf8=%E2%9C%93&search=TBARS&commit=Search&find_by_id= and https://aopwiki.org/events?utf8=%E2%9C%93&search=TBA&commit=Search&find_by_id=.

**4-HNE** is listed in AOP 149: Peptide oxidation leading to hypertension (status: under development, included in OECD Work Plan), link: https://aopwiki.org/aops/149 and AOP 260: CYP2E1 activation and formation of protein adducts leading to neurodegeneration (status: under development), link: https://aopwiki.org/aops/260, and in 2 KEs, link: https://aopwiki.org/events?utf8=%E2%9C%93&search=4-hydroxy-2-nonenal+&commit=Search&find_by_id=.

**3-Nitro-tyrosine** is mentioned in AOP 188: Iodotyrosine deiodinase (IYD) inhibition leading to altered amphibian metamorphosis (status: under development), link https://aopwiki.org/aops/188.

**COX2** is listed in 8 AOPs (3, 21, 43, 149, 150, 277, 293, 294), link: https://aopwiki.org/aops?utf8=%E2%9C%93&search=Cyclooxygenase+2&commit=Search&find_by_id= and in 4 KEs, link: https://aopwiki.org/events?utf8=%E2%9C%93&search=cyclooxygenase+2&commit=Search&find_by_id=.

**CAT** is listed in 5 AOPs (17, 37, 131, 148, 296), link: https://aopwiki.org/aops?utf8=%E2%9C%93&search=catalase&commit=Search&find_by_id= and in 3 KEs, link: https://aopwiki.org/events?utf8=%E2%9C%93&search=catalase&commit=Search&find_by_id=.

**SOD** is mentioned in 6 AOPs (17, 46, 48, 220, 296, 301), link: https://aopwiki.org/aops?utf8=%E2%9C%93&search=superoxide+dismutase+&commit=Search&find_by_id= and in 4 KEs, link: https://aopwiki.org/events?utf8=%E2%9C%93&search=superoxide+dismutase+&commit=Search&find_by_id=.

**GPx1** is listed in 4 AOPs (4, 119, 209, 271) and 5 KEs, link: https://aopkb.oecd.org/search.ashx?qry=erythrocyte+glutathione+peroxidase.

**GR** is listed in 7 AOPs (120, 124, 187, 209, 289, 305, 1573) and 12 KEs, link: https://aopkb.oecd.org/search.ashx?qry=glutathione+reductase+.

**TrxR** is mentioned in AOP 17: Binding of electrophilic chemicals to SH (thiol)-group of proteins and/or to seleno-proteins involved in protection against oxidative stress during brain development leads to impairment of learning and memory (status: under development, included in OECD Work Plan), link: https://aopwiki.org/aops/17 and is listed in 6 other AOPs (120, 124, 187, 289, 305, 1573) and 8 KEs, link: https://aopkb.oecd.org/search.ashx?qry=thioredoxin+reductase+&sort=0.

**GSH** is listed in 15 AOPs (3, 17, 31, 37, 46, 144, 148, 149, 205, 209, 220, 260, 293, 294, 296), link: https://aopwiki.org/aops?utf8=%E2%9C%93&search=glutathione&commit=Search&find_by_id=, 3 KEs, link: https://aopwiki.org/events?utf8=%E2%9C%93&search=glutathione&commit=Search&find_by_id=, and one other KE, link: https://aopwiki.org/events/130.

**Se** is listed in AOP 17: Binding of electrophilic chemicals to SH (thiol)-group of proteins and/or to seleno-proteins involved in protection against oxidative stress during brain development leads to impairment of learning and memory (status: under development, included in OECD Work Plan), link: https://aopwiki.org/aops/17 and AOP 284: Binding of electrophilic chemicals to SH (thiol)-group of proteins and/or to seleno-proteins involved in protection against oxidative stress leads to chronic kidney disease (status: under development), link: https://aopwiki.org/aops/284.
